# Characterization of the complete chloroplast genome of *Bletilla striata* (Orchidaceae: Bletilla), the herb in China

**DOI:** 10.1080/23802359.2019.1676177

**Published:** 2019-10-12

**Authors:** Hui Feng, Tianyi Cao, Hong Xu, Yihui Liu, Ying Han, Wei Sun, Yi Liu

**Affiliations:** a Hangzhou Red Cross Hospital, Hangzhou, PR China;; b Zhejiang Chinese Medical University, Hangzhou, PR China;; c Psychosomatic Department of Hangzhou Seventh People’s Hospital, Hangzhou, PR China

**Keywords:** *Bletilla striata*, Orchidaceae, herb, chloroplast genome, phylogenetic and evolutionary

## Abstract

Chloroplast (cp) genome sequences have been become a useful tool for phylogenetic and evolutionary studies in the herb. In this study, the complete cp genome of *Bletilla striata* was assembled. The complete cp genome is circular of 157,016 base pairs (bp) in length, which harbours a large single-copy region (85,821 bp), a small single-copy region (17,743 bp), and two inverted-repeat regions (each one of 26,726 bp). The cp genome of *B. striata* contains 139 genes, including 94 protein-coding genes (PCGs), 37 transfer RNA (tRNAs), and 8 ribosome RNA (rRNAs). The overall nucleotide content of the cp genome is A of 31.0%, T of 31.8%, C of 18.9%, and G of 18.3%, with a total AT content of 62.8% and GC content of 37.2%. However, the phylogenetic relationship analysis using maximum-likelihood (ML) method, which the result showed that the position of *B. striata* was situated as *Bletilla ochracea* in Orchidaceae. This study will be helpful for studies on the cp genetic engineering and medicinal herb value of Orchidaceae species in further.

*Bletilla striata* is known as hyacinth orchid or Chinese ground orchid and is a species of flowering plant in the orchid family Orchidaceae. Its tuber (named as Baiji in Chinese) is also an important and common medicinal herb in China (Feng et al. 2008). It native to Japan, Korea, Myanmar, and China. The Chinese Pharmacopoeia states that *B. striata* supports haemostasis, detumescence, and promotes recovery (Yamaki et al. 1992). Phytochemical research revealed that *B. striata* contains polysaccharides, bibenzyl, phenanthrene, dihydrophenanthrene, flavonoids, and phenolic compounds (Shi et al. 2017). In this study, the complete chloroplast (cp) genome of *B. striata* is presented and studied the phylogenetic relationship, which can set up the genomic data and use for clinical drug development of it further.

The specimen sample of *B. striata* was collected from Zhejiang Chinese Medical University in Hangzhou, Zhejiang, China (30.28N, 120.15E). The cp genome DNA of *B. striata* was extracted from the tissue of tubers using the modified CTAB method and stored in Zhejiang Chinese Medical University (No. SCMC-ZJU-TCM-02). The whole cpDNA was purified and fragmented using the TAKARA Next Ultra™ II DNA Library Prep Kit (Dalian and China; TAKARA), that the cpDNA was sequenced. Quality control was performed to remove low-quality reads and adapters using the FastQC version 0.11.8 (Andrews 2015). The cp genome was assembled and annotated using the MitoZ (Meng et al. 2019). The physical map of the cp genome was drawn using OrganellarGenomeDRAW version 1.3.1 (Greiner et al. 2019). The complete cp genome of *B. striata* was annotated and submitted to the GenBank of NCBI accession No. MK9355231.

The complete cp genome of *B. striata* is the circular DNA molecule which is 157,016 base pairs (bp) in size, which harbours a characteristic quadripartite structure with a large single-copy region (LSC) of 85,821 bp, a small single-copy region (SSC) of 17,743 bp and two inverted repeat regions (IRs) of 26,726 bp. The cp DNA of *B. striata* contains 139 genes, including 94 protein-coding genes (PCGs), 37 transfer RNA genes (tRNAs), and 8 ribosomal RNA genes (rRNAs). Here, 22 genes were found duplicated in each one of IR regions, including 10 PCG species (*rpl22, rps19, rpl2, rpl23, ycf2, ycf15, ndhB, rps7, rps12, a*nd *ycf68*), 8 tRNA species (*trnH-GUG, trnI-CAU, trnL-CAA, trnV-GAC, trnI-GAU, trnA-UGC, trnR-ACG,* and *trnN-GUU*), and 4 rRNA species (*rrn16, rrn23, rrn4.5,* and *rrn5*). The overall nucleotide content of the cp genome is Adenine (A) of 31.0%, Thymine (T) of 31.8%, Cytosine (C) of 18.9%, and Guanine (G) of 18.3%, with a total AT content of 62.8% and GC content of 37.2%.

The Maximum-Likelihood (ML) phylogenetic tree was generated using the amino acid sequences of 39 PCGs among 16 cp sequences in Orchidaceae by MEGA X (Kumar et al. 2018). Here, ML analysis was performed using the GTR model and 5000 bootstrap values replicate at each node. All of the nodes were inferred with strong support by the ML methods. The final ML phylogenetic tree was edited using the iTOL version 4.0 online web (https://itol.embl.de/) (Letunic and Bork 2016). The phylogenetic relationship analysis result showed that the position of *B. striata* was situated as *Bletilla ochracea* (NC_029483.1) in Orchidaceae (Figure 1). However, the cp genome of *B. striata* is very important to study this herb species and also can be useful for medicinal herb value and clinical drug development in further.

**Figure 1.**
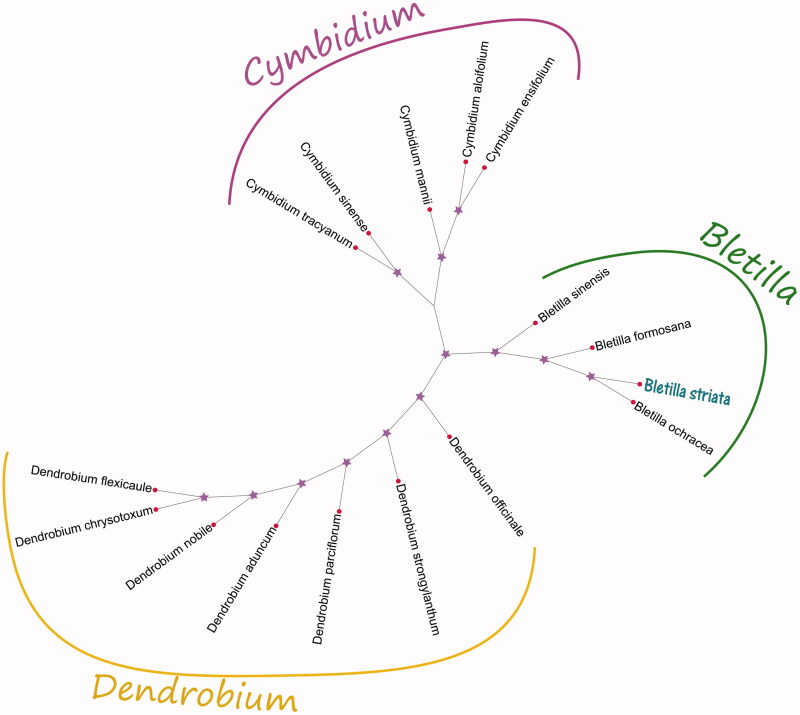
Phylogenetic of the amino acid sequences of 39 PCGs among 16 species within the family Orchidaceae based on the Maximum-Likelihood analysis of the complete cp genome sequences using 5000 bootstrap replicates. GenBank accession numbers: *Bletilla formosana KP866858.1, Bletilla ochracea NC_029483.1, Bletilla sinensis KP866862.1, Cymbidium aloifolium NC_021429.1, Cymbidium ensifolium NC_028525.1, Cymbidium mannii NC_021433.1, Cymbidium sinense NC_021430.1, Cymbidium tracyanum NC_021432.1, Dendrobium aduncum LC348859.1, Dendrobium chrysotoxum NC_028549.1, Dendrobium flexicaule LC348965.1, Dendrobium nobile KX377961.1, Dendrobium officinale KJ862886.1, Dendrobium parciflorum NC_035334.1,* and *Dendrobium strongylanthum NC_027691.1.*
